# Insights into the Sources, Structure, and Action Mechanisms of Quinones on Diabetes: A Review

**DOI:** 10.3390/molecules30030665

**Published:** 2025-02-03

**Authors:** Tingting Zhang, Mingyue Li, Jingyang Lu, Jia Wang, Min Zhang, Pharkphoom Panichayupakaranant, Haixia Chen

**Affiliations:** 1 Tianjin Key Laboratory for Modern Drug Delivery & High-Efficiency, School of Pharmaceutical Science and Technology, Tianjin University, Tianjin 300072, China; 17622636510@163.com (T.Z.);; 2 China-Russia Agricultural Processing Joint Laboratory, Tianjin Agricultural University, Tianjin 300384, China; 3 State Key Laboratory of Nutrition and Safety, Tianjin University of Science & Technology, Tianjin 300457, China; 4 Phytomedicine and Pharmaceutical Biotechnology Excellence Center, Faculty of Pharmaceutical Sciences, Prince of Songkla University, Hat Yai, Songkhla 90112, Thailand

**Keywords:** quinones, sources, types, antidiabetic effects, structure–activity relationship, activity mechanism, toxicity, bioavailability

## Abstract

Quinones, one of the oldest organic compounds, are of increasing interest due to their abundant presence in a wide range of natural sources and their remarkable biological activity. These compounds occur naturally in green leafy vegetables, fruits, herbs, animal and marine sources, and fermented products, and have demonstrated promising potential for use in health interventions, particularly in the prevention and management of type 2 diabetes (T2DM). This review aims to investigate the potential of quinones as a health intervention for T2DM from the multidimensional perspective of their sources, types, structure–activity relationship, glucose-lowering mechanism, toxicity reduction, and bioavailability enhancement. Emerging research highlights the hypoglycemic activities of quinones, mainly driven by their redox properties, which lead to covalent binding, and their structural substituent specificity, which leads to their non-covalent binding to biocomplexes. Quinones can improve insulin resistance and regulate glucose homeostasis by modulating mitochondrial function, inflammation, lipid profile, gastrointestinal absorption, and by acting as insulin mimetics. Meanwhile, increasing attention is being given to research focused on mitigating the toxicity of quinones during administration and enhancing their bioavailability. This review offers a critical foundation for the development of quinone-based health therapies and functional foods aimed at diabetes management.

## 1. Introduction

In recent years, with the growing global awareness of health and disease prevention, there has been an increasing focus on the use of natural products in health interventions, particularly in the prevention and management of metabolic diseases such as type 2 diabetes (T2DM) [1]. Modifying lifestyle and nutrient intake through health interventions using compounds from natural sources has become one of the core strategies used for public health [2]. Active ingredients from natural products, such as phytonutrients and natural antioxidants, have demonstrated a wide range of potential in the prevention and mitigation of chronic diseases due to their remarkable biological activities and pharmacological effects [3]. Quinones, in particular, have become an important factor in diabetes prevention and treatment research in recent years due to their unique biological activities and pharmacological effects.

Quinones of natural origin, such as pyrroloquinoline quinone (PQQ), vitamin K2 (VK2), and emodin, are widely found in vegetables, fruits, spices, herbs, animal and marine sources, and fermented products [4,5,6]. They are capable of binding to or interacting with various biological receptors, thereby eliciting a broad spectrum of pharmacological effects, including anticancer, antidiabetic, anti-inflammatory, antiarthritic, antifungal, antibacterial, antiviral, antiplatelet, and neuroprotective actions [7]. The diversity in substituents and their locations, as well as the redox properties of unsaturated quinone rings, including the electrophilic reactivity determined by the carbonyl group and the reaction of the polarized double bond with the nucleophile, all contribute to the peculiar chemical properties of quinones [8]. Quinones of natural origin have shown significant therapeutic potential in diabetes control. PQQ, known in the world of medicine as the “14th vitamin”, cannot be manufactured enough to meet the body’s needs by intestinal bacteria, and thus it must be supplemented from dietary sources such as fruits, vegetables, fermented foods, and breast milk [9]. Its hypoglycemic effects have been extensively researched [10]. PQQ demonstrates potential in alleviating streptozotocin-induced diabetes mellitus and mitigating oxidative stress in mice [11]. VK2 supplementation could alleviate impaired glucose homeostasis and insulin sensitivity in T2DM through gut flora and fecal metabolites [12]. One of the highest quality sources of VK2 is natto, which contains roughly 10 μg of menaquinone-7 per gram [13]. Cassia seed tea with a high anthraquinone content provides a range of health advantages, including antidiabetic, antibacterial, anti-inflammatory, hepatoprotective, and neuroprotective properties [14].

Diabetes mellitus, which has grown to be one of the most serious and common chronic metabolic diseases of our time, is characterized by hyperglycemia [15]. The incidence of diabetes has reached its peak globally due to factors such as a severely aging population and increased risk factors such as obesity due to poor dietary habits. The 10th International Diabetes Federation (IDF) report highlights diabetes as a rapidly growing global health emergency in the 21st century, with a prevalence of 10.5% (537 million adults) in 2021 and one death occurring every 5 s [16]. Over 90% of all cases of diabetes worldwide are caused by T2DM, which is the most prevalent kind of the disease and is brought on by insufficient insulin production or insulin resistance. Diabetes mellitus can reduce life expectancy and cause life-threatening, disabling, and costly complications [17], including microvascular complications, macrovascular complications, diabetic central nervous system complications, diabetic foot, and so on. And, in accordance with recent research, diabetes mellitus is also a significant risk factor for unfavorable outcomes in Coronavirus Disease 2019 (COVID-19). People with diabetes mellitus are substantially more likely than those without diabetes mellitus to require hospitalization and pass away from COVID-19 infection [18]. In recent years, scientists have been committed to multi-faceted and multi-level research, aiming to find more effective treatment drugs. Additionally, findings from the literature indicate that the percentage of undiagnosed diabetes is relatively high, particularly in low-income countries, where it may reach 50.5% in 2021 [16]. Therefore, interventions in daily life through improved dietary habits and the intake of natural nutrients are gradually being considered as effective strategies for coping with diabetes. Quinones of natural origin, however, are becoming an important component in the field of diabetes health intervention due to their wide range of sources and multiple biological effects.

Although natural quinones have shown great potential in the treatment and prevention of diabetes, there are still some obvious gaps and challenges in the existing research. First, the long-term safety and potential toxicity risks of quinones have not been adequately studied. Prone to toxic effects, quinones are one of the major reactive toxic metabolites in the body, accounting for about 40% of the total, including quinone-imines, quinone-methides, and imine-methides [19]. The hepatotoxicity of the hypoglycemic drug troglitazone has been attributed primarily to the formation of the active metabolite o-quinone methide [20]. Electrophilic quinoline derivatives resulting from the metabolism of nevirapine, a non-nucleoside human immunodeficiency virus -1 (HIV-1) reverse transcriptase inhibitor, could react with nitrogen-based nucleophiles to produce severe hepatotoxicity and serious adverse cutaneous reactions [21]. Therefore, the in-depth study and selection of quinones are of utmost importance.

Overall, natural quinones present novel opportunities for managing diabetes and its complications, especially in nutritional interventions and functional food development with a wide range of applications. Although current research indicates that quinones exhibit potential in regulating insulin signaling pathways, improving insulin resistance, and suppressing inflammatory responses, their specific mechanisms of action remain incompletely understood. Further exploration is needed, particularly regarding the molecular mechanisms underlying their ability to ameliorate insulin resistance and modulate glucose metabolism. Additionally, research on the bioavailability and metabolic pathways of quinones is insufficient, especially the pharmacokinetics and biotransformation processes in humans, which limits a comprehensive understanding of their clinical application. While natural quinones are generally considered relatively safe, data on their potential toxicity with long-term use and high doses, as well as their safety in specific populations (e.g., the elderly, pregnant women), remain lacking. Furthermore, studies on the application of quinones in functional foods have predominantly focused on the activity of single components, while their interactions and stability in complex food matrices are underexplored. Challenges in quality control and standardization, such as batch variability and compositional instability, may also affect their efficacy. This review will systematically summarize structural classification, natural sources, and mechanisms of action related to the anti-diabetic activity of natural quinones, with a focus on recent advances in bioactive quinones. It will also discuss the current research status and gaps in bioavailability, toxicity, and safety. By elucidating these research gaps, this review aims to provide a theoretical foundation for the application of natural quinones in the treatment of diabetes and its complications while promoting the development of dietary recommendations, development of supplements, or incorporation into pharmacological therapies and providing concrete suggestions for future research or clinical trials.

## 2. Types of Quinones

**Figure 1:**
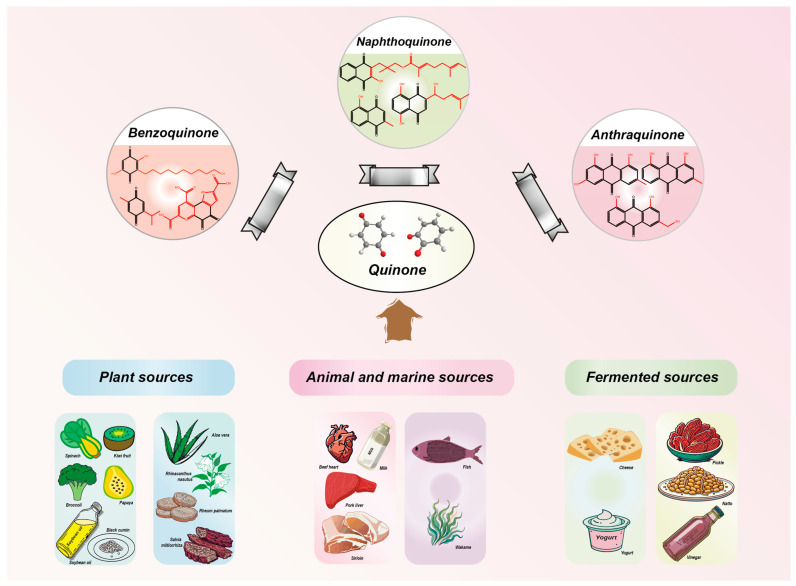
Main sources and types of quinones, three circles leading out of the silver banner represent the three types of quinones, and under the brown arrow are the natural sources of quinones.

Quinones are cyclohexadienediones that are divided into numerous types, namely benzoquinones, naphthoquinones, anthraquinones, and phenanthrenequinones, with their carbonyl (C=O) groups in either between the 1 and 2 positions or the 1 and 4 positions to one another [7]. The conformational flexibility and redox properties of quinones, such as their ability to participate in electron transfer reactions, play a critical role in their biological activity, particularly in modulating oxidative stress and mitochondrial function, which are key factors in T2DM [22,23]. Among these, benzoquinones, represented by coenzyme Q10 (CoQ10), demonstrate weaker hypoglycemic effects, but excel in antioxidant activity and cardiovascular protection [24]. This makes them particularly suitable for diabetic patients with cardiovascular comorbidities, although their low bioavailability and mild glucose-lowering effects often require combination therapy with other antidiabetic agents [25]. Naphthoquinones, such as rhinacanthin C (RC) and shikonin, also exhibit moderate hypoglycemic potential, primarily through the inhibition of α-glucosidase and the modulation of lipid metabolism [26,27]. While their ability to control postprandial glucose levels is notable, their cytotoxicity remains a significant limitation, restricting their broader clinical application. On the other hand, anthraquinones, such as emodin and chrysophanol, have been extensively studied for their strong hypoglycemic effects. They improve insulin sensitivity and β-cell function through multi-target mechanisms, including the activation of insulin signaling pathways and inhibition of enzymes like PTP1B and DPP-4 [8]. Additionally, their anti-inflammatory and antioxidant properties make them promising candidates for managing diabetes and its complications. However, concerns about hepatotoxicity and gastrointestinal side effects necessitate further research to ensure their safety [28]. Phenanthrenequinones, however, have received limited attention in hypoglycemic research due to their non-negligible toxicity. Consequently, most research efforts have focused on the first three types of quinones (Figure 1). Table 1 lists representative quinones with hypoglycemic potential, highlighting their diverse mechanisms and therapeutic profiles. Overall, while quinones offer promising avenues for diabetes management, further clinical studies are essential to validate their long-term safety and optimize their therapeutic applications.

### 2.1. Benzoquinones

Benzoquinone is the quinone with the simplest structure, having a benzene ring and two carbonyl groups. There are many classical natural quinones, such as p-benzoquinone CoQ10 and o-benzoquinone PQQ, both of which are widely available and have been intensively studied for their hypoglycemic activity. In the brains of streptozotocin-induced diabetic mice, PQQ might ameliorate oxidative stress and lipid peroxidation [10]. Embelin, a possible hypoglycemic drug with the ability to decrease lipid profiles, suppress intracellular pro-inflammatory mediators, and improve oxidative stress, was extracted from *E. basal* berries [32]. Thymoquinone, the predominant constituent of *Nigella satica* volatile oil, exhibited antidiabetic effects through its action on the serum glucose and insulin levels and body weight of the animals [30]. Genus *Ardisia* is the largest genus in the family Myrsinaceae. Twenty-eight benzoquinones have been identified from several *Aldisia* plants, including *Ardisia gigantifolia*, *Ardisia quinquegona*, *Ardisia kivuensis*, *Ardisia virens*, and *Ardisia japonica* [41]. Based on the substituents on the quinone ring, these quinones could be categorized into belamcandaquinones and alkylated benzoquinones.

### 2.2. Naphthoquinones

Both vitamin K1 (VK1) and VK2 are classical menaquinones. In one Danish Diet Cancer and Health study, VK1 intake was found to be linearly inversely associated with incident diabetes in all subgroups [42]. VK2 supplementation enhanced glucagon-like peptide (GLP-1) levels in circulation, colon bile acid receptor activation, and amelioration of host immune-inflammatory responses in diet-induced obese mice [12]. The main active ingredients of *Rhinacanthus nasutus* are naphthoquinones. Up to now, more than twenty-six naphthoquinones have been isolated and identified from *Rhinacanthus nasutus*. The nomenclature of naphthoquinones is characterized by “rhinacanthin”. Rhinacanthin-rich extract (RRE) could be an immunomodulatory agent, regulating the levels of interleukin 1beta (IL-1β), interleukin-6 (IL-6), and tumor necrosis factor-alpha (TNF-α) [26]. Furthermore, RRE successfully restored normalcy to the levels of protein that were causing hemoglobin A1c (HbA1c), insulin resistance and β cell dysfunction (homeostasis model assessment-insulin resistance (HOMA-IR) and homeostasis model assessment β cells (HOMA-β)), serum levels of total cholesterol (TC), total triglycerides (TG), high-density lipoprotein (HDL), and low-density lipoprotein (LDL), key enzymes related to liver function (aspartate aminotransferase (AST) and alanine aminotransferase (ALT)), serum blood urea nitrogen (BUN), and creatinine linked to kidney function [43]. Among them, RC showed the highest hypoglycemic activity. In addition to *Rhinacanthus nasus*, there are other tropical plants that are also rich in hypoglycemic naphthoquinones, such as *Kigelia africana*, *Diospyros kaki*, *Plumbago zeylanica*, *Impatiens balsamina*, and *Lawsonia inermis* [35]. Plumbagin, the primary naphthoquinone found in *Plumbago zeylanica*, could increase the release of insulin from β-cells, potentially having antidiabetic effects at doses of 15 or 30 mg/kg [44].

### 2.3. Anthraquinones

Common hypoglycemic anthraquinones of plant origin, such as emodin, chrysophanol, aloe-emodin, physcion, and rhein, are biosynthesized through the polyketide pathway or the chorismate/O-succinylbenzoic acid pathway [45]. In 3T3-L1 adipocytes, emodin had a favorable impact on glucose metabolism, which might have been mediated by an increase in glycolysis through the adenosine monophosphate-activated protein kinase (AMPK) signaling pathway [40]. Aloe-emodin could protect RIN-5F cells from nuclear morphology and DNA damage and modulate the levels of pro-inflammatory cytokines [46]. Chrysophanol was a competitive protein tyrosine phosphatase 1b (PTP1B) inhibitor, and its combination with emodin could suppress PTP1B activity and strongly enhance insulin sensitivity in high-fat diet (HFD)-fed mice [47]. Physcion isolated from *Senna obtusifolia* could strongly inhibit PTP1B activity (IC_50_, 7.28 μM) [48]. Rhein isolated from *Rheum rhizome* and *Rheum emodi* could promote glucose uptake in both Min6 cells and 3T3-L1 cells [8]. *Reynoutria japonica*, *Rheum palmatum*, *Cassia obtusifolia*, and *Pleuropterus multiflorus* are typical herbs with anthraquinones as the dominant active ingredients [49]. Studies have been conducted to compare these four herbs. The results of the comparison of anthraquinone content were *Rheum palmatum* > *Cassia obtusifolia* > *Reynoutria japonica* > *Pleuropterus multiflorus*, and the results of the comparison of total free anthraquinone content were *Rheum palmatum* > *Reynoutria japonica* > *Cassia obtusifolia* > *Pleuropterus multiflorus*. So different herbs might have different activities based on the different quinone types and contents.

## 3. Sources of Quinones

Natural sources of quinones are extremely diverse (Figure 1), including vegetables, fruits, and herbs from plant sources, meat, eggs, and dairy products from animal sources, and fish, shellfish, and algae from marine sources. In addition, fermented products such as natto and yogurt are equally important sources of quinones. These natural products are playing an increasingly important role as part of a healthy green treatment, especially in the management of T2DM. Studies have shown that an appropriate increase in the intake of quinone-rich natural products, especially substances such as VK1 and VK2 and CoQ10, can significantly improve glycemic control and insulin sensitivity. Specifically, VK1-rich foods were strongly associated with a reduced risk of developing diabetes, and this was further validated by multivariate-adjusted Cox proportional hazards modeling [42]. Primary dietary sources of VK2 in humans include dairy products as well as fermented vegetables, which have been found to improve impaired glucose homeostasis and insulin sensitivity [12,50]. Meat, fish, nuts, and certain oils represent the most abundant dietary sources of CoQ10, which may help control blood glucose, lower total cholesterol, and improve high-density lipoprotein cholesterol (HDL-C) in patients with T2DM [24,51]. Thus, a green health intervention strategy based on natural quinones not only provides a new perspective on the integrated management of type 2 diabetes, but also highlights the potential of naturally sourced products in diabetes control. By emphasizing the diversity and complementarity of natural products, this health intervention approach not only focuses on dietary optimization, but also advances overall health management strategies.

### 3.1. Plant Sources

In line with efforts to valorize agricultural by-products, researchers have found many vegetables, fruits, and oils to be valuable dietary sources of quinones. The specific contents of some quinone sources can be found in Table 2. High PQQ content was found in vegetables, such as parsley (34.2 ± 11.6 ng/g), green pepper (28.2 ± 13.7 ng/g), and spinach (21.9 ± 6.19 ng/g), and fruits such as Kiwi fruit (27.4 ± 2.64 ng/g) and papaya (26.7 ± 8.57 ng/g) [52]. Dietary sources of VK1 (phylloquinone) are mainly vegetables (56.5–76.8%) [53]. Typical foods include spinach, kale, broccoli, lettuce, cabbage, and other green leafy vegetables [5]. Spinach contains 240–1220 µg/100 g of K1 and kale contains 250–1139 µg/100g [50]. VK1 is relatively stable during cooking, and boiling and microwaving have little effect on its content [50,54]. Vegetable oils (e.g., 180 μg/100 g of soybean oil, 130 μg/100 g of rapeseed oil, and 55 μg/100 g of olive oil) are considered to be the second largest contributor of VK1 to the human diet [54,55]. VK1 is fat-soluble, and the absorption of VK1 is enhanced when other VK1-related foods are consumed with vegetable oil [50]. Additionally, there has been a lot of attention in recent years on the use of spices for the treatment of numerous ailments and their beneficial effects on health. Referring to the USDA’s FoodData Central database, although spices are not a major source of VK1 like green vegetables, some of these herbs such as sage (*Salvia officinalis*), thyme (*Thymus vulgaris*), basil (*Ocimum basilicum*), coriander leaf (*Coriandrum sativum*), parsley (*Petroselinum crispum*), marjoram (*Origanum Majorana*), etc., still contain significant amounts of VK1 [56]. One of the representative active ingredients of black cumin (*Nigella sativa*) seeds is one quinone compound, thymoquinone, which accounts for about 18–24% of volatile oil components. Black cumin supplementation of 2 g/day for three months was administered to 114 individuals with T2DM in one clinical study, which markedly improved glucose homeostasis and reduced fasting blood glucose (FBG) and HbA1c levels [57].

At the same time, quinones are also found in numerous medicinal plants, which are often used in tea culture to achieve appropriate therapeutic effects. Certain beverages were found to have extremely high levels of VK1, including green powdered tea (28.54–32.44 μg/g), natural leaf tea (tea leaves) (17.58–19.94 μg/g), and black tea (tea leaves) (9.45–11.27 μg/g), and it is vital to note that there was almost no VK1 in some brewed tea beverages, such as brewed natural leaf tea and brewed black tea [58]. Quinones are present in a wide variety of plant families, such as Ranunculaceae, Asphodelaceae, Fabaceae, Ebenaceae, and Rhamnaceae [62]. Quinones have been isolated and identified from fruits [32], flowers [63], nuts [64], seeds [48], stems [41], leaves [64], roots [65], and whole plants [35]. In contrast, the presence of quinone in leaves and roots has been studied more. Aloe-emodin, initially derived from *Aloe vera* leaves, prevented glucotoxicity in RIN-5F cells by controlling pro-inflammatory cytokines [46]. Lawsone, a significant specialized 1,4-naphthoquinone synthesized from 1,4-dihydroxy-2-naphthoate (DHNA), was responsible for the reddish-orange dye extracted from the leaves of *Lawsonia inermis* [64]. Both the roots and the leaves of *Rhinacanthus nasutus* contain RC; however, the roots have a higher concentration [66]. Administration of RC at doses of 5 mg/kg/day or 20 mg/kg/day for 28 days notably enhanced FBG, HbA1c, insulin, and lipid levels in male streptozotocin-nicotinamide-induced diabetic rats [67]. Quinone diterpenes are abundant in the Salvia species, mainly in the roots, and a total of 175 abietane quinone diterpenes with o-quinone and p-quinone chromophores were detected in 130 species of salvia [63]. Anthraquinones isolated from the roots of *Knoxia valerianoides* effectively inhibited the formation of advanced glycation end products (AGEs) and rat lens aldose reductase in vitro [68].

### 3.2. Animal and Marine Sources

Animals are also important natural sources of quinones, mainly benzoquinones and naphthoquinones and their derivatives. Fish and many tissue types of meat such as heart, liver, shoulder, sirloin, thigh, tenderloin, etc., are very rich sources of dietary CoQ10, with heart being the most prominent (beef heart, 113.3 mg/kg; pork heart, 118.1–282 mg/kg; chicken heart, 92.3–192 mg/kg; herring heart, 120.0–148.4 mg/kg; mackerel heart, 105.5–109.8 mg/kg) [51]. Even though breast milk (140–180 ng/mL) contains dozens of times more PQQ and its derivatives overall than the average meal, it has not been proven that higher organisms can independently synthesize PQQ [59]. Butter as a dairy product is rich in VK2 (15 μg/100 g) [5]. Tert-butylhydroquinone (TBHQ) is an antioxidant widely used in animal-derived foods and oils, which could elevate insulin resistance and liver steatosis through adenosine monophosphate-activated protein kinase alpha 2 (AMPKα2)/phosphoinositide 3-kinase (PI3K)/protein kinase B (AKT) signaling in diabetes [69,70]. Quinones have also been found in some echinoderms, a class of marine invertebrates [62]. Many algae, such as wakame and laver, contain higher levels of VK1 than terrestrial plants [60]. Sesquiterpene quinones and their derivatives, recognized as significant bioactive metabolites, are commonly found in the sponges of the genus *Dysidea* [71]. They are usually not directly edible, but can be used as medicine. 21-Dehydroxybolinaquinone, derived from Hainan sponge *Dysidea villosa*, has been demonstrated to inhibit the activity of hPTP1B, a prospective therapeutic target for the treatment of T2DM and obesity [72]. Additionally, ascidians of the genus *Aplidium* are regarded as a significant source of naturally occurring, chemically varied, and physiologically active compounds [73]. Anthraquinones, such as altermodinacid A, emodic acid, and emodin, were also found in some marine fishes due to the co-existence of fungi such as a halotolerant fungus *Alternaria* sp. X112 isolated from *Gadus macrocephalus* [74].

### 3.3. Fermented Sources

Microbial communities are the most widely distributed in nature. Through phytophagy, commensalism, and parasitism, they have tight ties to other creatures, including animals, plants, and microbes, and are crucial components of the biosphere and ecosystems [75]. Microorganisms are non-negligible sources of quinones and are the main force behind the mass production of many quinones, such as some filamentous fungi like *Aspergillus*, *Penicillium*, *Talaromyces*, *Fusarium*, *Arthrinium*, etc. [76]. Emodin may be found in the fungi *Aspergillus*, *Cladosporium*, *Chaetomium*, *Penicillium*, and *Penicilliosis* as a red pigment [6]. Wijesekara extracted physcion from the *Microsporum sp.* Fungus [77]. Microbial processing of dietary products can be seen everywhere in daily life. *Aspergillus* is an important strain in the fermentation and food processing industries such as the production of sauces, brewing, and vinegar, and nearly 60 strains have been utilized [78]. *Penicillium* is the core ingredient of the famous blue cheese [79]. However, little research has been performed to date on the direct correlation between the nutrition or flavor of foods fermented by these genera and the existence of quinones. In contrast to VK1, which is primarily present in fruits and vegetables, VK2 is more commonly found in bacteria, such as the gut microbiota, where it can be found in the bacterial membrane [80]. Our daily intake of vitamin K needs to be 1 μg per kilogram of body weight [81]. *Lactobacilli* are one representative strain that produce VK2 (534 nmol/g VK2 of lyophilized cells) and are considered starter cultures for food products with a Generally Recognized As Safe (GRAS)-approved safety profile [81]. *Lactobacillus*-derived dietary resources are abundant and include common cheese dairy products, yogurt dairy products, and some fermented vegetables such as tempeh and pickles. There are also research data that support the factual nature of the high VK2 content in fermented products: natto (10 μg/g menaquinone-7) [13], cheese (parmesan, 7.1–76.5 μg/100 g; emmental, 43.3 μg/100 g; gouda, 47.3–72.9 μg/100 g) [5], and pickle (1.00–4.63 µg/100 g menaquinone-4, 5.55–14.48 µg/100 g menaquinone-7) [82]. PQQ is a coenzyme that resembles a vitamin, and PQQ disodium salt has received significant attention as a brand-new nutritional supplement in China, the European Union, and the United States. The PQQ’s biosynthesis in higher organisms has not been demonstrated, so humans rely mainly on dietary intake, with the primary source of production thought to be microorganisms. PQQ-producing strains are mainly Gram-negative bacteria, and their biosynthetic process is mainly catalyzed by multiple proteins encoded by the PQQ gene cluster [83]. The PQQ content of fermented soybeans (natto, 61.0 ± 31.3 ng/g; green soybeans, 9.26 ± 3.82 ng/g) was significantly higher [52]. It has been demonstrated that certain foods, such as vinegar, which are associated with acetic acid bacteria, contain relatively high concentrations of PQQ [84]. In the future, more fermented resources rich in quinones, which are potentially biologically active, may be identified.

## 4. Role of Quinones’ Physicochemical Properties in Diabetes Control

The chemistry of quinones bears many similarities to those of α-β unsaturated ketones. A significant portion of their redox characteristics stem from their electrophilic reactivity, which is dictated by the carbonyl groups, and their interaction with nucleophiles through polarized double bonds [85]. They interact with biological systems as a class of highly reactive organic chemical species, promoting anti-inflammatory, antibacterial, antimalarial, anti-diabetic, and anticancer actions and inducing toxicities.

Quinones often use non-covalent or covalent binding to enzymes or biological complexes to achieve their functions [61]. The polarity of the quinones influences the degree of cellular uptake and subsequent binding across the lipid membrane to the corresponding target to produce an active effect. Anders hypothesized that the more lipophilic quinones would stay in the lipid layer for a longer period of time, and, due to the higher solubility of oxygen in the lipid layer, quinones with an inherently lower reduction potential may also predict a rapid electron transfer to oxygen (O_2_) [85]. Differences in chirality affect activity, with dextro-sennidin A promoting adipocyte glucose incorporation more than sennidin B [86]. The glycosylation of compounds can also cause changes in activity; for example, increased glycosylation tends to reduce the inhibitory effect of rhubarb phenols on PTP1B [48].

The physicochemical properties essentially influence quinones’ overall biological activities (functional, toxic, mutagenic, or therapeutic effects) and their metabolism, which is a comprehensive book of quinone pharmacology and toxicology [28]. What follows is an analysis of which chemical properties affect the hypoglycemic activity of quinones in two main aspects: redox reactions, and non-covalent interactions.

### 4.1. Role of Quinones in Redox Reactions

Quinones have excellent hypoglycemic activity, but they also produce toxic effects, so quinones are regarded as a double-edged sword that acts as a hypoglycemic agent and treats diabetes. The biological activity and toxicity of quinones are strongly influenced by the redox properties of quinones, both in terms of their prooxidant action as electron transfer agents and in terms of their addition-reduction reactions as electrophiles to bind biological complexes [62]. One quinone can undergo multiple redox transformations and undergo different biotransformations at the same time.

#### 4.1.1. Quinones as Electron Transfer Agents

The role of quinones in glucose-lowering interventions is closely related to their unique redox properties. As electron transfer agents, quinones are able to participate in free radical chain reactions, which in turn affect reactive oxygen species (ROS) levels in the body. The extent to which these reactions are carried out determines the antidiabetic effect of quinones and their potential toxicity. Under appropriate conditions, quinones can exert their hypoglycaemic effects by modulating oxidative stress and promoting insulin sensitivity. PQQ could ameliorate oxidative stress and lipid peroxidation in the brains of streptozotocin-induced diabetic mice [10]. Chrysophanol ameliorates oxidative stress and pyroptosis in mice with diabetic nephropathy by modulating the Kelch-like ECH-associated protein 1/nuclear factor erythroid 2-related factor 2 signaling pathway [87]. However, elevated ROS levels could induce deoxyribonucleic acid (DNA) damage, disrupt the DNA damage response (DDR), and lead to lipid peroxidation, as well as protein binding, which may alter their conformation and function [88]. Thus, there is an exquisite line of equilibrium between the antidiabetic effects of quinones and their toxicity.

**Figure 2:**
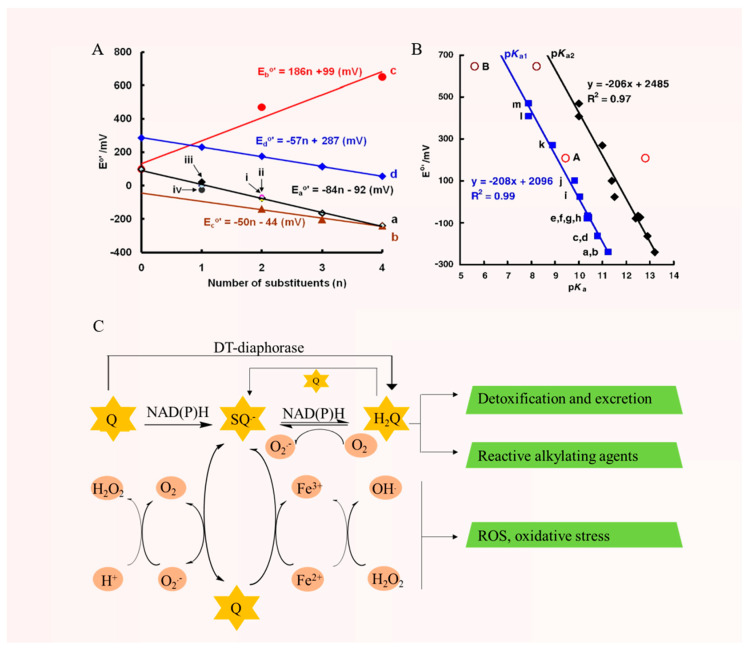
Quinones as electron transfer agents. (**A**) Substituents influence E°′(Q/SQ^−^) and E°′(Q, 2H^+^/H_2_Q) of quinones [90], (a) E°′(Q/SQ^•−^) vs. number of alkyl groups on the benzoquinone ring, (b) E°′(Q/SQ^•−^) vs. number of methyl groups on 1,4-naphthoquinone, (c) E°′(Q/SQ^•−^) vs. number of chlorines on benzoquinone, (d) E_2_°′(Q, 2H^+^/H_2_Q) vs. number of methyl groups on benzoquinone. (**B**) E°′(Q/SQ^•−^) correlates with the p*K*_a’_s of the corresponding para-hydroquinones [90]. The squares (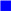, blue) are the first p*K*_a_ of a particular hydroquinone and the diamonds (♦, black) are the second p*K*_a_ of the hydroquinone. (**C**) Redox cycling of quinones, the brown triangles represent various structural variations of the quinone, while the red circles indicate key factors involved in the reaction process.

The free radical chain reaction of quinone can be seen in Figure 2C. Quinone (Q) can be reduced by enzymes, mostly flavoproteins, such as cytochrome P450 (CYP450) reductase, ubiquinone oxidoreductase, cytochrome b5 reductase, etc., through two one-electron reductions, first to semiquinone (SQ^•−^) and then to hydroquinone (H_2_Q) [89]. nicotinamide adenine dinucleotide phosphate (NADPH) or nicotinamide adenine dinucleotide hydride (NADH) is used as the electron source to provide the electrons for the reaction. Under aerobic conditions in the body, SQ^•−^ can be rapidly autoxidized to form Q, accompanied by the production of superoxide radical anion (Q_2_^•−^), due to the higher content of oxygen compared to quinone. The redox cycle of quinones may continue with the accumulation of Q_2_^•−^ up to anaerobic levels, followed by an increase in the accumulation of SQ^•−^, and the whole cycle is prone to oxidative stress and cell damage [28]. H_2_Q can undergo autoxidation in the presence of oxygen, and it has been shown that it can react with O_2_ to produce quinone and hydrogen peroxide (H_2_O_2_) [90]. However, subsequent studies have shown that the autoxidation of H_2_Q is not limited to this reaction, as H_2_Q can react with O_2_ to produce SQ^•−^ and Q_2_^•−^. And SQ^•−^ can further react with oxygen to produce quinone and Q_2_^•−^, or with Q_2_^•−^ to produce H_2_O_2_. In addition, H_2_Q can react with Q to produce SQ^•−^ by one disproportionation reaction. In terms of reaction kinetics, H_2_Q is more likely to undergo a disproportionation reaction with quinone [91]. One important reason why quinones can undergo redox cycling is that the quinone/quinol ring remains intact during the redox reaction [90]. Hydroquinone can also be produced directly by a two-electron reduction through DT-diaphorase, e.g., NADPH: quinone oxidoreductase, a common two-electron reductase. Xanthine oxidase, lipoamide oxidoreductase, and xanthine dehydrogenase catalyze both one- and two-electron reduction. Direct two-electron reduction to produce hydroquinone has traditionally been considered a quinone detoxification mechanism [92]. If a water-soluble and stable H_2_Q is produced, it can be conjugated with glucuronides or sulfates and excreted. Unstable hydroquinone can undergo chemical rearrangement, leading to the alkylation of vital biomolecules (e.g., DNA), or engage in redox reactions that generate highly reactive oxygen species [93].

The whole redox process is governed by the reduction potential of the parent quinone, namely the reduction potential Q/SQ^•−^ and SQ^•−^/H_2_Q, with the reduction potential Q/SQ^•−^ dominating overall. A more positive reduction potential facilitates the redox reaction, while a lower reduction potential for Q/SQ^•−^ promotes the reduction in oxygen to form Q_2_^•−^ and quinone via SQ^−^. At the same time, this reaction is reversible, and the reduction potential of oxygen (O_2_/Q_2_^•−^) is −180 mV [94]. Therefore, according to the reduction potential of quinones, it can be judged whether this quinone compound is a reactive ROS scavenger or an ROS producer. If the reduction potential Q/SQ^•−^ is higher than −180 mV, the consumption of O_2_^−^ by quinone is thermodynamically favorable [90]. In addition, it has been proposed that if Q/SQ^•−^ is higher than −150 mV, the reaction rate constant of SQ^•−^ with oxygen follows the electron transfer of Marcus theory well, while below −150 mV, the rate constant is affected by diffusion [95]. The substituents on the quinone ring affect its electron density of the quinone, which in turn affects the reduction potential. If the electron donating group (−CH_3_, −OCH_3_) is substituted, the reduction potential will decrease, and if the electron withdrawing group (−Cl, −COOH) is substituted, the reduction potential will increase [96]. Figure 2A shows that, with the increase in alkyl substitution of benzoquinone and 1,4-naphthoquinone, the negative charge of single-electron reduction potential increases, and the reduction potential shows a linear decrease, whereas an increasing the substitution of chlorine atoms on benzoquinone leads to a linear increase in the reduction potential [90]. The substituent position of quinone compounds is particularly important. Studies have shown that the prooxidant action of lawsone with a hydroxyl substituent on the quinone ring is weaker than that of juglone with a hydroxyl substituent on the benzene ring, and juglone has stronger toxic effects than lawsone [97]. The p*K*_a_ value of hydroquinone is also a reflection of the degree of electron density, which is proportional to the p*K*_a_ value of semiquinone, and is also somewhat indicative of the reduction potential as well as the kinetic rate constant. With the substitution of electron-giving groups on the quinone ring, H_2_Q has a higher p*K*_a_ value, which is inversely proportional to the reduction potential of quinone and is more likely to drive the oxidation of SQ^•−^ to generate Q_2_^•−^ and more likely to drive the autoxidation of H_2_Q [90] (Figure 2B). Moreover, the autoxidation of H_2_Q is pH-dependent; the higher the pH, the faster the reaction rate [98]. The reaction rate increases in the presence of metal ions, and the rate of radical production is greater than the rate of radical elimination, which predisposes to oxidative stress. Q_2_^•−^, driven by metals, tends to trigger a cascade reaction, leading to the production of H_2_O_2_ and hydroxyl radical (OH^•^) [28], which is considered the most active ROS (Figure 2C). In addition, one general trend in reduction potential is o-quinone > discrete quinone > p-quinone, e.g., 1,2-naphthoquinone (68 mV) > 1,4-naphthoquinone (50 mV) [90,96]. The higher the reduction potential, the easier it is to be reduced. The reduction potential is also to some extent related to molecular weight, and the reduction potential decreases with the increase in molecular weight, e.g., 1,4-benzoquinone (99 mV) > 1,4-naphthoquinone (50 mV) > 9,10-anthraquinone (−445 mV), 1,2-naphthoquinone (68 mV) > 9,10-phenanthrenequinone (−124 mV) [90,96].

In conclusion, quinones exhibit antidiabetic potential in regulating the generation and scavenging of ROS through their redox properties. Their efficacy and safety are highly dependent on a combination of factors such as their reduction potentials, molecular structures, environmental conditions, and metal ion concentrations. Understanding these influencing factors can help to better balance the glucose-lowering effects of quinones with the risk of toxicity and provide more options for health interventions in diabetes management.

#### 4.1.2. Quinones as Electrophiles

Another important redox property of quinones is that they are prone to the Michael addition reaction of nucleophiles to form covalent bonds due to their electrophilic α- and β-conjugated systems [62]. However, in general, the carbon atom next to the quinone group is required to have no substituent, so anthraquinones generally do not undergo the Michael addition reaction [28]. Substitution of 2-hydroxy-1,4-naphthoquinone and 2-amino-1,4-naphthoquinone in the quinone ring at position 3 eliminates nephrotoxicity due to the blocking of the Michael addition reaction [99]. The addition of quinones as electrophiles is affected by substituents, electron symmetry, and polarity [28]. As mentioned earlier, substituents of the quinones affect the reduction potential of the quinone. A lower reduction potential of quinone correlates with a higher p*K*a value of hydroquinone, indicating reduced electrophilicity [90], and it is more difficult to react with affinity reagents and the reaction rate is slower. Moreover, the steric hindrance of substituents on quinones reduces the reactivity of quinones, as does the increased density of electron clouds on quinones [100]. The affinity reagents for quinone binding mainly include amines (the −NH_2_ part of free amino acids, the amino groups on lysine and arginine, or the amino groups on DNA bases) and thiols (e.g., glutathione). In comparison, there is a 10^8^ rate difference between the reaction with thiols and the amine reaction, and the content of thiols in the body is higher, so thiols are the main reaction objects [90]. In general, the generated thioether hydroquinone has an increased p*K*a value, which can undergo autoxidation, produce free radicals, cause oxidative stress, or bind to DNA and proteins, directly affecting their structure and efficacy [85]. Sulfhydryl groups in cysteine are the predominant redox-active and nucleophilic functional groups within biological systems. This amino acid is integral to various proteins, including the redox-active peptide glutathione, a tripeptide composed of gamma-glutamyl–cysteine–glycine. Glutathione functions as a reducing agent and nucleophile, protecting cells from chemical damage that could induce stress. Cysteine thiols also play a vital role as nucleophiles in the catalytic centers of numerous enzymes, including those involved in hypoglycemic effects, the glycolytic enzyme (Glyceraldehyde-3-phosphate dehydrogenase (GAPDH)), PTP1B, and kelch-like ECH-associated protein 1 (Keap1) [28].

### 4.2. Role of Quinones in Non-Covalent Interaction

**Figure 3:**
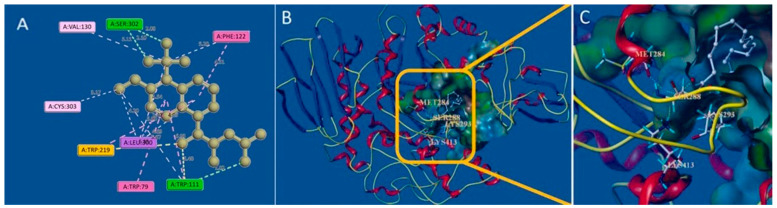
Role of quinones in non-covalent interaction. (**A**) Molecular docking between tolrestat and aldose reductse [38]. (**B**,**C**) Docking binding model of one embelin derivative with yeast α-glucosidase, the active site MOLCAD surface representation of lipophilic potential [103].

The non-covalent binding of quinones to biological complexes such as protein targets is usually low-energy and reversible and includes hydrophobic interactions, hydrogen bonding, van der Waals forces, and electrostatic interactions (Figure 3). When hydrophobic interactions dominate, the thermodynamic properties tend to be free energy greater than or equal to 0 (ΔH ≥ 0) and entropy greater than 0 (ΔS > 0), and the binding of small molecules to proteins is an entropy-driven process, whereas when hydrogen bonding and van der Waals forces dominate, the thermodynamic parameters have free energy less than 0 (ΔH < 0) and entropy less than 0 (ΔS < 0), and binding is enthalpy-driven [101]. Currently, the analysis of small molecule-protein binding is becoming increasingly sophisticated, not only in terms of experimental characterization methods involving equilibrium dialysis, isothermal titration calorimetry, size exclusion chromatography and affinity chromatography, nuclear magnetic resonance spectroscopy, small-angle scattering techniques for X-rays and neutrons, and mass spectrometry [102]. In addition, with the development of theoretical calculations, computerized molecular simulation techniques have been widely used in the field of pharmacological activity research, which lay a theoretical foundation for experimental exploration to a certain extent.

Many quinones exhibit their hypoglycemic potential through hydrophobic binding, hydrogen bonding, and electrostatic interaction with target proteins. Important non-protein electron carriers in the mitochondrial respiratory chain of animals and the photosynthetic chain of plants, such as CoQ10 and plastoquinone, are characterized by their long side chain structures [104]. At the same time, some studies have found that insulin receptors have corresponding hydrophobic binding pockets, and the length of hydroxyalkyl chains on quinones affects their efficacy [105]. In the study of the glucosidase inhibitory activity of embelin derivatives, the two hydroxyl groups and the long-chain substituents were the key factors affecting the inhibitory activity. Molecular docking results show that hydrogen bonds were formed between the hydrophilic group on the active site and several amino acids, and the hydrophobic interaction between the hydrophobic substituent at position 3 and the hydrophobic pocket of benzoquinone was an important inhibitory mechanism [103]. Glycosylated hydroquinones undergo halogenation on the benzene ring, which can promote glucose phosphorylase. The hydroxyl group of hydroquinone was methylated, and the activity was significantly increased. According to the results of molecular docking, the inhibitor stabilized the enzyme’s low-active T state by means of a complex network of van der Waals interactions with residues of the 280 s loop (Asn283 and Asn284), glycine helix (Gly134 and Gly137), and Glu88, as well as direct and water-mediated hydrogen bonds and halide interactions. These forces stabilized the conformation of the 280 s loop and organized glycogen entry [106].

## 5. Mechanisms of Anti-Diabetic Activity of Quinones

**Figure 4:**
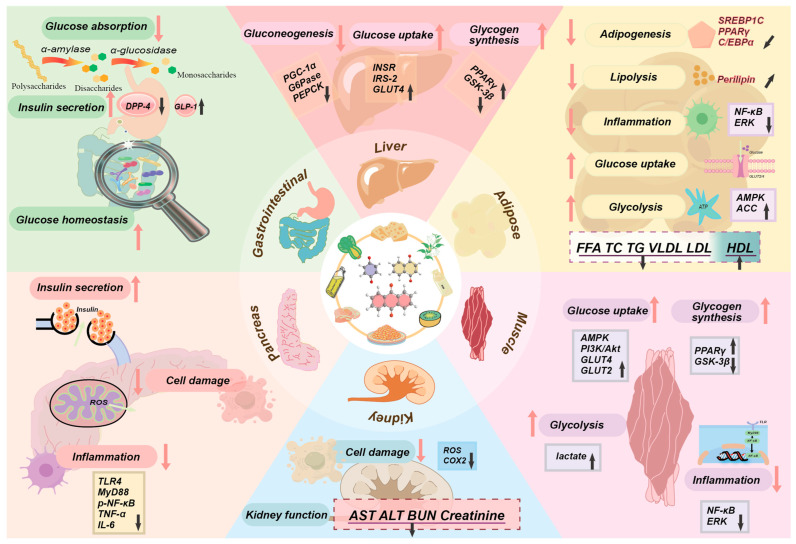
Regulation of glucose homeostasis in different organs by quinones (the information in the figure is summarized in the article description and Table 1 information.).

As an important part of endocrine homeostasis, diabetes affects the physiological homeostasis of all tissues and organs. Correspondingly, the preventive and therapeutic effects of quinones are found in all parts of the body and are inseparably interrelated. Many quinones have been reported to have dose-dependent hypoglycemic effects, such as thymoquinone [107]. Quinones show different mechanisms for the regulation of glucose homeostasis in different organs (Figure 4). The main mechanisms of the hypoglycemic effect of quinones are described in detail below.

### 5.1. ROS Adjustment and Mitochondrial Homeostasis

**Figure 5:**
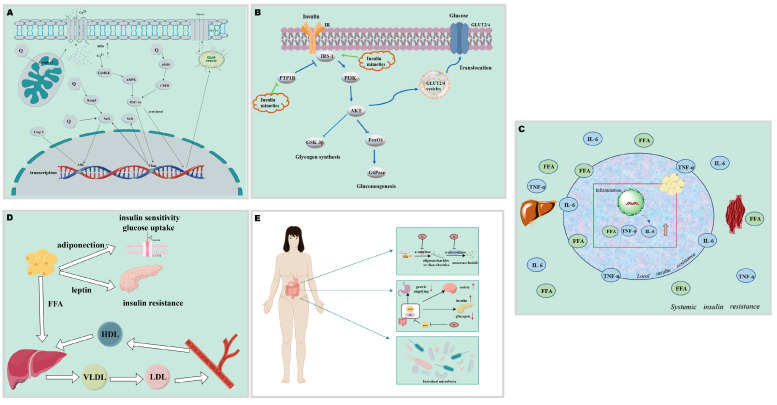
Regulation of glucose homeostasis by a variety of mechanisms (partly drawn by Figdraw (https://www.figdraw.com/#/)). (**A**) ROS adjustment and mitochondrial homeostasis. (**B**) Insulin mimetics. (**C**) Regulation of inflammation. (**D**) Regulation of lipid profile. (**E**) Regulation of gastrointestinal absorption.

Natural quinones can reduce insulin resistance to achieve a hypoglycemic effect by regulating ROS level and mitochondrial function balance (Figure 5A). In T2DM, hyperglycemia and insulin resistance are observed, with studies indicating that approximately 10% of consumed oxygen may be lost in the form of ROS [108]. This phenomenon is closely associated with the critical role of oxidative stress in the pathogenesis of diabetes. Oxidative stress primarily arises from an imbalance between pro-oxidants, such as ROS, and antioxidants, including catalase (CAT) and superoxide dismutase (SOD) [109]. When ROS levels become excessive and antioxidants are unable to effectively neutralize them, the cellular redox balance is disrupted, leading to cellular damage and dysfunction. This imbalance not only exacerbates the oxidative stress response, but also impairs mitochondrial function. Mitochondria are the primary source of ROS within cells, and while they generate energy through oxidative phosphorylation, they also release substantial amounts of ROS in the process [110]. In mitochondria, elevated redox potential causes changes in the oxidative state, leading to the uncoupling of oxidative phosphorylation, a process that inhibits the efficiency of the electron transport chain. This results in an inhibition of electron transport and an increase in the transfer of electrons to oxygen, subsequently leading to the formation of O_2_^−^ [31]. Since mitochondrial complex I is the primary rate-limiting site in the electron transport chain, it has become a critical target in diabetes research. When quinones bind to mitochondrial complex I, studies show that they induce conformational shifts that affect its function, which may impact mitochondrial activity and oxidative phosphorylation [111]. It has also been shown that during the action of quinones on the mitochondrial respiratory complex, NADH-induced changes can only occur in the open conformation of the complex, whereas quinone reduction can only occur in the closed state, with proton pumps involved in the transition between the two states [112]. This conformational shift may lead to the dysfunction of complex I, thereby impacting the entire mitochondrial energy metabolism and ROS production.

In the treatment of diabetes, the activation of AMPK is considered an important regulatory pathway. AMPK could improve insulin sensitivity by promoting glucose uptake, glycolysis, and fatty acid oxidation while also inhibiting glycogen and protein synthesis [113]. These effects make AMPK a potential target for the treatment of diabetes. Emodin could promote AMPK activation and stimulate glucose uptake in a ROS-calmodulin-dependent protein kinase kinase (CaMKK)-dependent manner [114]. PQQ has been found that it could facilitate the ’recoupling’ of endothelial nitric oxide synthase (eNOS) and mitochondrial oxidative phosphorylation, stimulate phosphorylation and the activation of the Cyclic Adenosine Monophosphate (cAMP) response element-binding protein (CREB), and enhance the expression of the peroxisome proliferator-activated receptor-gamma coactivator-1 alpha (PGC-lα) [4]. Elevated levels of PGC-lα binded to and co-activated the transcriptional function of nuclear respiratory factor (Nrf-1/2) at the Tfam promoter, which was essential for the regulation of mtDNA amplification and mitochondrial biogenesis. Nrf2:INrf2 (Keap1) function as cellular sensors of oxidative and electrophilic stress [115]. And anhydroexfoliamycin isolated from Streptomyces could protect cells from oxidative stress, improve mitochondrial function, act on the Nrf2-ARE pathway, inhibit the effect of the mitochondrial uncoupler carbonyl cyanide-p-trifluoromethoxyphenylhydrazone (FCCP) over cytosolic Ca^2+^, and reduce caspase-3 activity [116]. In experimental studies, oxidative stress is generally judged by measuring the expression levels of glutathione (GSH), SOD, CAT, malondialdehyde (MDA), and glutathione peroxidase (GPx). Reactive aldehyde MDA is one of the reactive electrophiles known to produce toxicity [117]. Increased oxidative stress and free radical production may be compensated for by elevated levels of GSH and SOD [32]. The activity of the peroxisome-based enzyme CAT, which converts hydrogen peroxide into water and oxygen, is increased in the diabetic rats’ erythrocytes, heart, aorta, kidney, and liver [118]. GSH and GSH/GPx enzyme systems are essential for normal intracellular homeostasis, which could become disrupted in pathophysiologic conditions, such as endothelial dysfunction [119]. Many quinones, such as embelin, RC and emodin, were found to have a modulating effect on oxidative stress, with decreased levels of MDA and increased expression of SOD, CAT, MDA, and GPx [8,32,34]. These quinones could act by enhancing the expression of antioxidant enzymes and improving mitochondrial antioxidant capacity, ultimately reducing ROS levels and mitigating the deleterious effects of oxidative stress in diabetes. In conclusion, natural quinones show promising effects in modulating oxidative stress and mitochondrial dysfunction, which are key factors in diabetes. However, further clinical studies and experimental data are required to better understand their precise mechanisms and therapeutic potential in diabetes management.

### 5.2. Insulin Mimetics

According to modern research, insulin mimetics have two main mechanisms of action (Figure 5B): (1) activation of the tyrosine kinase structural domain of IR, specifically IRS-1, which causes autophosphorylation of the receptor and thus the activation of downstream signaling pathways that are essential for the metabolic action of insulin, and (2) the inhibition of tyrosine protein phosphatases, which dephosphorylate IR and IRS, thus impeding the insulin signaling pathway [120].

Demethylasterriquinone B1 (DAQ B1), a symmetrical bisindolequinone natural compound, triggered the insulin receptor tyrosine kinase at concentrations of 3–6 μM, inducing phosphorylation of insulin [121]. And it has been found to promote glucose uptake in rat adipocytes and mouse soleus muscle in ob/ob mice, which might be associated with its capacity to phosphorylate IR-β and then initiate the PI3K/AKT/glucose transporter protein 4 (GLUT4) pathway. 5,8-diacetyloxy-2,3-dichloro-1,4-naphthoquinone (DDN) was also a molecular activator for the insulin receptor, which could be used in combination with insulin acting in the extracellular domain and DDN acting in the intracellular domain [122]. Emodin improved insulin sensitivity and alleviated insulin resistance by increasing hepatic glucose utilization and muscle and fat glucose uptake through the IRS/PI3K/Akt/forkhead box O 1 (FoxOl) pathway [123].

As a member of the PTPs family, PTP1B negatively regulated the insulin and leptin signaling pathways [124]. According to Kennedy et al., mice with the PTPIB gene lacking showed higher insulin sensitivity. Insulin treatment in these mice produced enhanced and extended phosphorylation of the insulin receptor in the liver and muscle tissue, indicating that PTPIB was critical in initiating the dephosphorylation of insulin receptors [125]. As a result, it was identified as a crucial target for the treatment of obesity and T2DM. Ertiprotafib and trodusquemine were two representative small molecule PTP1B inhibitors currently in development [126]. With an IC_50_ value of 6.70 μM, dysidine was a new slow-binding PTP1B inhibitor that activated the insulin signaling pathway and facilitated GLUT4 translocation. In 3T3-L1 cells, it has been demonstrated to stimulate a 2.3-fold increase in glucose uptake [72]. PQQ could activate insulin signaling independently of the ligand through redox cycling reactions, and the redox product hydrogen peroxide catalyzed the oxidative modification of cysteine to inhibit PTP1B activity [4].

Analysis of the PTP1B inhibition by 1,2-naphthoquinone derivatives revealed the importance of the substituent at the R4 position [126]. Additionally, it has been shown that, independent of the linkage site, the inhibitory effect of anthraquinones on PTP1B was enhanced by an increase in the amount of hydroxyl groups [8]. Hydroxyl groups on R1 or R4 of anthraquinones were essential for upsetting a number of intracellular signaling pathways, according to research by Fukuda et al. [127]. Jung et al. found that chrysophanol’s glycosylation tended to reduce PTP1B inhibition, with chrysophanol > chrysophanol-8-O-β-D-glucopyranoside > chrysophanoltriglucoside > chrysophanol fourraglucoside as the sequence of glycosylation [48]. This refined understanding of quinones, particularly those targeting the insulin receptor and PTP1B, underscores their potential as therapeutic agents in T2DM treatment, while also highlighting key structural elements that influence their bioactivity and efficacy. Further studies are required to optimize these compounds for clinical application, especially in terms of improving their bioavailability and ensuring long-term safety in chronic treatment settings.

### 5.3. Regulation of Inflammation

Inflammation in the onset and course of diabetes plays a causal and potentially innate role (Figure 5C). The release of pro-inflammatory factors in the immune system can lead to insulin resistance. Pro-inflammatory factors contribute to the transition from normal to impaired glucose homeostasis in adipose, liver, pancreatic, and skeletal muscle cells, particularly in the obese condition [128]. Due to its limited capacity for storing energy, adipose tissue accumulates excess calories and eventually experiences hypoxia. This activates hypoxia-inducible factor-1 (HIF-1), which then causes increased expression of c-Jun N-terminal kinase (JNK) and IκB kinase (IKK), which in turn causes the release and accumulation of inflammatory cytokines and free fatty acid (FFA), ultimately resulting in insulin resistance, ectopic fat deposition, and an imbalance in the production of adipokines [128]. Activated or inflammatory macrophages may interfere with the functioning of pancreatic β-cells. The cytokine IL-1β can impair insulin secretion and promote Fas expression, leading to Fas-triggered islet cell apoptosis [129]. M1-polarized inflammatory macrophages within kupffer cells are affected by FFA as well as cytokines in vivo, which not only reduce glucose uptake in skeletal muscle, but also induce insulin resistance in the liver [130]. The pro-inflammatory factor IL-6 can cause insulin resistance in skeletal muscle as well as the liver due to defects in IRS phosphorylation [131]. TNF-α has antagonistic activity against insulin because of its ability to enhance phosphorylation of IRS on serine or threonine residues. This leads to a reduction in tyrosine phosphorylation of protein kinase C (PKC), nuclear factor kappa B (NF-κB), and kappa B kinase β (IKKβ), thereby affecting downstream signaling pathways, including fatty acid regulation, glucose uptake, and triglyceride synthesis [32]. Additionally, it has been emphasized how important intestinal inflammation is for the development of diabetes [132].

Recent studies on benzoquinones, such as those derived from the roots of *Averrhoa carambola* L., highlight their potential anti-inflammatory effects. These compounds have been shown to reduce the expression of pro-inflammatory cytokines like TNF-α and IL-6 in pancreatic tissue, as well as downregulate the toll-like receptor 4 (TLR4)/NF-κB pathway [65]. Rhein reduced the expressions of inflammatory markers (TNF-α and IL-6), hexosamine, and mitogen-activated protein kinase p38 pathways, and could potentially prevent oxidative complications induced by diabetes [133]. Furthermore, emodin safeguarded RINm5F cells and mouse islets from cytokine-induced damage by inhibiting inducible nitric oxide synthase expression and reducing nitric oxide production [134]. This cytoprotective effect may be linked to inhibition of NF-κB signaling through a reduction in inhibitor of kappa B (IκB) kinase activity. These findings suggest that quinones had the potential to modulate inflammation in diabetes, offering a promising therapeutic strategy to mitigate insulin resistance and other disease complications.

### 5.4. Regulation of Lipid Profile

Diabetes is significantly linked to changes in the plasma lipid profile (Figure 5D). These disturbances in lipid metabolism, characterized by increased levels of TG and FFA, contribute to pancreatic β-cell dysfunction, induce insulin resistance in various organs, and exacerbate the overall metabolic imbalance [135]. LDL and very low-density lipoprotein (VLDL) transport cholesterol to peripheral tissues, where it is deposited, while HDL facilitates the transfer of cholesterol from peripheral tissues to the liver, promoting its excretion and metabolism [32]. Elevated serum levels of LDL and VLDL are hallmark indicators of the initiation of the atherosclerotic process, which is often exacerbated in diabetic individuals, increasing their risk for cardiovascular diseases [136]. Leptin and adiponectin, which are also secreted by adipocytes, have been associated with the development of insulin resistance. Plasma leptin/adiponectin ratio (LAR) is inversely correlated with insulin sensitivity indexes [137]. Specifically, leptin levels are negatively correlated with insulin sensitivity, with elevated leptin contributing to insulin resistance [138]. In contrast, adiponectin could regulate Rho/ROCK-dependent actin cytoskeleton remodeling, thereby enhancing glucose uptake and metabolism [139]. Embelin, a compound derived from the plant *Embelia ribes*, has demonstrated promising effects on diabetes management. Embelin has been shown to enhance insulin secretion in pancreatic β cells while concurrently reducing the levels of TC, TG, LDL, and VLDL and increasing the expression of HDL in the bloodstream [32]. Additionally, administration of RC to diabetic rats led to an increase in the atherogenic index (LDL/HDL ratio), HOMA-IR, and HOMA-β cell function index, thereby ultimately reducing the risk of cardiovascular complications associated with diabetes [67]. These changes ultimately suggested a reduced risk of cardiovascular complications typically associated with diabetes. Furthermore, emodin, another bioactive compound, has been found to significantly alter lipid and inflammatory markers in diabetic mice. In KKAy mice, a diabetic model, treatment with emodin led to substantial changes in lipid profiles, including reductions in TC, triglycerides (TAG), and LDL-C, while simultaneously increasing HDL-C levels. Moreover, emodin intervention resulted in lowered FFA levels and reduced inflammatory markers such as high-sensitivity C-reactive protein (hs-CRP) and TNF-α [123]. These findings highlight the potential of emodin as a therapeutic agent not only for improving glucose metabolism, but also for ameliorating the dyslipidemia and inflammation that accompany diabetes. In summary, the interplay between the lipid metabolism, insulin resistance, and the cardiovascular risks associated with diabetes underscores the importance of managing lipid profiles alongside blood glucose levels. Compounds like embelin and emodin show potential in targeting these metabolic disturbances, suggesting their possible therapeutic roles in improving insulin function, reducing atherosclerotic risk, and modulating inflammation in diabetic patients. Further clinical investigations are needed to confirm their efficacy and safety for long-term use in diabetes management.

### 5.5. Regulation of Gastrointestinal Absorption

The regulation of gastrointestinal absorption is a significant approach in the treatment of T2DM with quinones (Figure 5E). Intake of food begins with the breakdown of amylase into small molecules. Many current hypoglycemic studies focus on the inhibition of amylase activity, with the aim of specifically forming inhibitory complexes with α-amylase in the mouth and intestines to block the breakdown and absorption of starch. By delaying the breakdown of dietary disaccharides or oligosaccharides into absorbable monosaccharides, α-glucosidase inhibition decreases glucose uptake and lowers blood sugar levels [8]. By analysis of α-glucosidase inhibition on embelin derivatives, it is found that the hydroxyl group at the second or fifth position of p-benzenquinone was important, and the long chain substituents at the third position were preferred [103]. Hydrogen bonds formed between the hydrophilic group of the active site and several amino acids and the hydrophobic interaction between the hydrophobic substituent at position three of p-benzoquinone and the hydrophobic pocket, which was long and relatively narrow, were important mechanisms of inhibition. RC demonstrated good α-glucosidase inhibition, and studies of RC interaction at the enzyme binding site show that alkaline residues around the naphthone ring helped bind the more electronegative parts of the enzyme [140]. Emodin and its derivatives, such as aloe-emodin, alaternin, and questin, were isolated from *S. obtusifolia* L. and *R. emodi* Wall. These compounds exhibited significant inhibitory activity against α-glucosidase, with IC50 values of 1.02 μM for emodin, 1.40 μM for aloe-emodin, 0.99 μM for alaternin, and 136.19 μM for questin [8]. And it was found that the number of hydroxyl groups had an impact on α-glucosidase inhibition, with the hydroxyl group at the R1 position being particularly important [8]. The presence of methoxy was shown to be important, as physcion inhibited α-glucosidase more than chrysophanol [48]. In most cases, glycosylation resulted in increased activity of obtusifolin, while chrysophanol showed decreased activity [48].

The inhibitory impact of dipeptidyl peptidase-4 (DPP-4) can raise the glucagon-like peptide 1 (GLP-1) and gastric inhibitory peptide (GIP) levels in the intestine, which in turn can alter the islet cells to decrease glucagon release and boost the cAMP level in the islet cells. This conveys signals during protein phosphorylation, delays gastric emptying, and reduces food intake by central appetite restriction [8]. GLP-1, secreted by intestinal cells, plays a crucial role in regulating blood glucose levels by promoting insulin secretion and inhibiting glucagon release. Emodin, which was found in *R. palmatum* L., was shown to effectively suppress DPP-4 activity with an IC_50_ value of 5.76 μM. However, the binding affinity of emodin to DPP-4 protein at Glu205 and Glu206 sites was relatively low [141]. Chrysophanol was observed to bind to the active site of DPP-4 with a docking score of −5.67 kcal/mol and to glibenclamide with a docking score of −3.9 kcal/mol, forming interactions through five hydrogen bonds. These findings indicated that chrysophanol had the potential to act as a DPP-4 inhibitor [142].

Intestinal microbiota is implicated in various physiological processes, including nutrition, substance metabolism, immune function regulation, and inflammatory response. Dysbiosis within the intestinal microbiota can adversely impact the metabolism of energy and substances, induce inflammatory responses, and result in metabolic disorders such as diabetes. Increased studies have shown that a high-fat diet and intestinal flora imbalance can weaken the intestinal mucosal barrier, thus increasing the body’s inflammatory response, inducing insulin resistance, and promoting the occurrence and development of diabetes. Aloe-emodin, rhein, and emodin, as effective components of Guanxin Xiaoban capsules, could effectively inhibit the advanced glycation end products–receptor for advanced glycation end products (AGE-RAGE) signaling pathway, and this mechanism seemed to be related to changes in intestinal flora, with an increase in the proportion of Akkermansia and a significant decrease in the proportion of Faecalibaculum [143]. Some studies have found that menaquinones were the main growth factors of bacteria in the human intestinal microbiota, including Faecalibacterium, Bacteroides, Bilophila, Gordonibacter, and Sutterella [144]. Dietary vitamin K was remodeled by intestinal flora and affected the flora composition in male and female C57BL6 mice [80].

The analysis highlights the intricate mechanisms of gastrointestinal regulation through quinones, specifically their impact on digestive enzyme inhibition, intestinal microbiota modulation, and DPP-4 activity, all of which are significant in the management of T2DM. Further research focusing on the pharmacokinetics and bioavailability of quinone compounds is essential to fully understand their therapeutic potential and optimize their use in diabetes treatment.

## 6. Toxicity

### 6.1. Quinone Toxicity in Metabolism

Quinones, such as VK1, VK2, CoQ10, rhodopsin, and components of certain plant-based ingredients, have complex chemical structures that are both biologically active and potentially toxic. With the widespread use of these compounds in nutritional and medicinal supplements, it has become increasingly important to study their toxicity and effects on human health. The cytotoxicity and biological activity of quinones, however, are not uniform and can vary significantly depending on their specific chemical structure. For instance, a study on p-benzoquinone congeners revealed that the most cytotoxic quinones were those with the highest electron affinity and the smallest molecular volume [145]. They are capable of accepting and transferring electrons and participating in redox reactions that both provide cellular protection and may, in some cases, lead to cellular damage and toxicity. Quinones have the potential to induce oxidative stress by generating free radicals, which can cause damage to cellular membranes and DNA [28]. At appropriate doses, quinones usually have favorable pharmacological effects and are less toxic. However, excessive doses or prolonged use can upset this balance and increase the risk of toxicity, potentially triggering hepatotoxicity, nephrotoxicity, and genotoxicity [146]. This “dose-dependent duality” is the central paradox in assessing its safety. Both 1,4-naphthoquinone and 1,2-naphthoquinone demonstrated substantial genotoxicity and cytotoxicity. In contrast, benzo[a]anthracene-7,12-quinone exhibited high genotoxicity and cardiovascular toxicity, and 9,10-phenanthrenequinone displayed negligible genotoxicity but nearly the highest cytotoxicity [147]. It has been demonstrated that chrysophanol, emodin, dantron, and aloe-emodin were mutagenic in bacteria, mostly through frameshift mutations and DNA damage [28]. 2,3-Dimethoxy-1,4-naphthoquinone (DMNQ) has the ability to cause hepatotoxicity, which in turn can be intensified by CYP450 inhibition [148]. Meanwhile, studies on the toxicity of emodin have shown that emodin has testicular reproductive toxicity due to the disruption of testicular gene expression [149].

Although plant-derived quinones show potential in the management of T2DM, the risks of their long-term use need to be critically weighed. *Frangula purshiana*, known for its anthraquinone content, particularly emodin, could inhibit insulin aggregation and fibrillation, potentially improving insulin function in diabetic models [150]. However, chronic use raised concerns about gastrointestinal toxicity, including melanosis coli and potential colorectal cancer risks [151,152]. Similarly, *Senna alexandrina* contained sennosides that demonstrated hypoglycemic effects; however, long-term consumption was associated with severe adverse reactions such as kidney and liver toxicity, gastrointestinal hemorrhage, and other complications [151]. *Aloe vera* was recognized for its glucose-lowering effects due to its polysaccharides and anthraquinones. Nevertheless, chronic use could lead to diarrhea, hypokalemia, and even carcinogenicity linked to whole leaf extracts [153]. Overall, while quinones may offer therapeutic benefits for T2DM management, their long-term safety remains a critical concern that requires further investigation to ensure that the risks do not outweigh the benefits in chronic treatment settings.

The toxicity of quinones is not limited to exogenous uptake; their pathways as endogenous metabolites are equally critical. Quinones are an active metabolite in the body that cannot be ignored. Many drugs, foods, or inhalants entering the body can be metabolized by the body’s metabolic enzyme system to produce quinones, which are highly reactive and easily bind to biological macromolecules in the body. Hypoglycemic drugs, such as troglitazone, are catalyzed by enzymes such as CYP450 and peroxidase to produce troglitazone quinone, which is prone to hepatotoxicity [20]. It has also been found that the observed toxicity of phenols can be correlated with the properties of their orthoquinone oxidation products or metabolites [154]. In addition, inhalants such as air pollutants, including fossil and diesel combustion and tobacco smoke, contain significant amounts of polynuclear aromatic hydrocarbons, such as naphthalene, which can be metabolized to quinones in the body and produce physiological toxicity [89].

### 6.2. Strategies to Reduce Quinone Toxicity

To reduce the toxicity of quinones, recent studies have begun to explore co-administration, structural modifications, and novel delivery systems. By combining quinones with other antioxidants or anti-inflammatory components, the oxidative stress and inflammatory responses they provoke are reduced. CoQ10 and sitagliptin co-administration for the treatment of diabetic nephropathy showed better renoprotective effects than CoQ10 or sitagliptin given alone [155]. By modifying the chemical structure of quinones, compound forms that retain their pharmacological activity while reducing toxicity are explored. Sulfonation of anthraquinones may reduce their toxicity because of their enhanced polarity, decreased cellular permeability, and decreased capacity to attach to DNA molecules to form complexes [28]. And it has been studied to achieve the goal of controlled masking and targeted release of redox cycling through the C-C bond-cleaving 1,6-elimination of orthoquinones [156]. At the same time, delivery systems such as nanoparticles and hydrogels can be used to achieve the targeted delivery of drugs, resulting in more targeted and less toxic therapeutic regimens. The use of Pluronic F-127 to encapsulate emodin in a hydrogel to create a topical delivery system improved the solubility of emodin while greatly reducing the liver injury associated with systemic emodin administration [157]. Nanoparticles encapsulating chrysophanol have been shown to improve its bioavailability and augment its nephroprotective properties [158].

### 6.3. Artificial Intelligence and Computational Approaches in Toxicology

Studies on the toxicity of quinones, which are potentially toxic due to their complex structure, are particularly critical in medicinal safety. The combination of modern technologies, including artificial intelligence and advanced computational methods, with toxicological studies represents a major paradigm shift. This shift has increased the speed, efficiency, and accuracy of toxicological evaluations, allowing for researchers to address complex questions about food safety exposures. High-throughput screening techniques enable the rapid assessment of the toxic effects of a large number of compounds, and, in combination with advanced tools such as mass spectrometry and nuclear magnetic resonance, scientists can accurately analyze chemicals and their metabolites in food at the molecular level [159]. Such techniques significantly reduce the time required for traditional toxicity assessments and provide more detailed insights into the metabolic pathways of chemicals in the human body and their interactions with biological systems. Recent developments in deep learning algorithms have proven effective for constructing quantitative structure–activity relationship (QSAR) models aimed at predicting toxicity. The models leverage large datasets to enhance the accuracy of toxicity predictions, enabling researchers to rapidly assess potential hazards associated with chemical compounds [160]. Computer-aided predictive modeling, including the application of computational platforms such as Discovery Studio, ProTox-II, Derek Nexus, ChemSpider, VEGA QSAR, Tox21 Data Challenge, SimCYP, etc., has significantly improved the efficiency of food safety assessments. Hughes et al. offer a rapid screening mechanism for critical drug toxicity risk with their simulation of quinone formation [19]. The model had an area under the receiver operating characteristic curve (AUC) accuracy of 97.6% for predicting pairs of atoms forming quinones and an AUC of 88.2% for identifying molecules forming quinones. Such high precision underscores the potential of AI-driven tools in toxicity assessments. At the same time, the integration of toxicology with genomics and bioinformatics has further deepened the understanding of the complex interactions between chemical substances and biological systems through gene expression and molecular dynamics analyses [161]. Toxicogenomics uses gene expression analysis to reveal the molecular mechanisms of toxicity, providing a theoretical basis for more accurate toxicity assessments. In summary, the application of modern technology, especially artificial intelligence, big data, and predictive modeling, has enabled toxicological studies to more efficiently and accurately address the assessment of potentially hazardous substances, especially the toxicity of complex structures such as quinones, which is of great significance in enhancing medicinal safety.

### 6.4. Research Challenges and Future Directions of Quinone Toxicity

In the realm of diabetes management, quinones found in various foods have shown promise in enhancing glucose metabolism [162,163]. However, their use presents potential toxicity risks, particularly with long-term consumption. To address these risks, regular blood tests to monitor liver and kidney function are essential for early toxicity detection [164]. Additionally, controlling the dosage and duration of quinone intake, especially for anthraquinones that may cause gastrointestinal issues, is crucial [165]. Personalized medicine approaches, based on individual genetic profiles, can further tailor treatment plans to minimize toxicity [166]. Encouraging the intake of quinones from natural food sources, which may have lower toxicity due to accompanying protective compounds, is another effective strategy. Adherence to established safety guidelines and education of patients and healthcare providers are vital for early intervention. Lastly, exploring adjunct therapies that enhance quinone benefits while reducing toxicity can offer a safer and more effective management approach [167,168]. By implementing these strategies, healthcare providers can safely leverage the benefits of quinones in diabetes management while minimizing associated risks.

Toxicity studies on quinones are still in a deepening stage. Despite their significant biological activity, the potential toxicity risks of these compounds cannot be ignored. Current studies have revealed the dual nature and complexity of quinones; however, the understanding of their mechanisms of toxicity, long-term exposure risks, and individual differences is incomplete. Research on natural product-based health interventions should shift focus from isolated active components to a more holistic approach, emphasizing the synergistic effects of various bioactive compounds found in these natural sources. The complexity of interactions among multiple compounds within natural products—such as herbs, fruits, and other herbs—could play a significant role in the prevention and management of chronic diseases [169]. Current studies have mostly focused on single quinones, overlooking the potential synergistic or antagonistic effects of their co-ingestion as part of a broader natural product intake. In addition, individual metabolic differences are influenced by genetic, health status, and environmental factors, and exposure risks are under-assessed, especially for specific populations with lower metabolic efficiency, where toxicity risks may be higher [170]. However, most studies have been limited to healthy adults and lack comprehensive assessments of the elderly, children, or diseased groups. Existing toxicity studies rely mainly on in vitro experiments and animal models, but extrapolation to humans is limited due to species differences and complex metabolic pathways in the human body. Assessments of short-term or single-dose exposures ignore the risk of chronic toxicity from long-term low-dose intake. Quinones can accumulate over long periods of time and participate in body metabolism, which may pose potential health risks. In addition, the stability of quinones during food or herb processing and the formation of their toxic derivatives under high-temperature conditions still lack in-depth studies. The development of modern technologies, especially the application of big data analysis, artificial intelligence, and computational modeling, has provided new tools for toxicity studies and is expected to optimize the toxicity assessment process. However, future studies still need to strengthen the investment in human trials, develop more sophisticated in vivo and ex vivo simulation models, and conduct comprehensive assessments of long-term exposure risks in order to reveal more accurately the health effects of quinones and their safety limits.

## 7. Bioavailability

Quinones have significant biological activities in food and drugs, especially in the prevention and treatment of metabolic diseases such as diabetes, which show great potential. However, the problem of bioavailability of quinones has been a major obstacle limiting the research and application of their pharmacological effects. Bioavailability refers to the extent to which an active ingredient is effectively utilized by the body during digestion and absorption and is influenced by factors such as solubility, absorption rate, metabolic pathway, and distribution in the body [171]. Some important quinones, such as rhodopsin, VK1/2, PQQ, and CoQ10, despite their good hypoglycaemic activity, usually have low bioavailability due to their poor water solubility, rapid metabolism, and excretion, limiting their clinical applications.

Bioavailability of oral PQQ appears to be low in several body systems [172]. The concentration of PQQ in human serum reached its peak 3 h post-administration, with its clearance profile in serum closely mirroring that observed in urine [173]. Although PQQ demonstrates high redox cycling capacity, its hydrophilic nature and rapid systemic clearance result in suboptimal pharmacokinetics. To improve absorption, formulations integrating PQQ into food matrices or liposomal carriers have shown promising results, enhancing its intestinal uptake and stability in circulation [168]. PQQ undergoes minimal enzymatic modification in the body and is excreted in its intact form through urine. It is metabolically stable, with its primary role being redox cycling, which supports mitochondrial function and reduces oxidative stress, beneficial for glucose regulation in T2DM [173]. CoQ10 is converted to its reduced form, ubiquinol, by NAD(P)H-dependent quinone reductases in tissues. Ubiquinol acts as a potent antioxidant, neutralizing free radicals and improving mitochondrial function in T2DM patients [174]. Its metabolism is highly dependent on age and individual mitochondrial activity. Due to its pronounced hydrophobicity and substantial molecular weight, CoQ10 exhibits slow and inefficient absorption in the gastrointestinal tract, with oral bioavailability typically remaining below 5% [25]. Recent advancements in formulation technologies, such as nanoemulsions, phytosomes, and ubiquinol-based formulations, have improved CoQ10’s solubility and absorption. Nanoemulsions have increased its bioavailability by up to threefold, ensuring higher plasma concentrations and better therapeutic efficacy [167]. The bioavailability of vitamin K has also been the focus of research. VK1 has a low dietary absorption rate of approximately 5–15% and a short half-life [53]. VK1 is more readily absorbed from vegetable oils than from vegetables. VK1 is capable of self-renewal through redox cycling in the liver and can be reused many times, which explains its lower daily requirement [53]. In contrast, Vitamin K2, particularly MK-7, has superior bioavailability due to its extended isoprenoid chain, slower clearance, and higher systemic retention. MK-7 also exhibits a longer half-life, allowing for better distribution in tissues and sustained therapeutic effects [175]. Novel emulsification techniques have further enhanced the bioavailability of Vitamin K2. Vitamin K is metabolized in the liver via reduction to its active hydroquinone form, which plays a key role in γ-carboxylation reactions critical for glucose metabolism and vascular health [176]. Anthraquinones also show good hypoglycaemic activity, e.g., rhein, emodin, chrysophanol, and aloeemodin, which are mainly absorbed in the intestine. It was found that the order of bioavailability of some anthraquinones is rhein > emodin > chrysophanol > aloeemodin [177]. Due to their high lipid solubility, free anthraquinones are absorbed at a faster rate than conjugated glycosides. The fluctuating blood concentrations and multiple absorption peaks of anthraquinones may be related to hepatic and intestinal circulation and reabsorption. Anthraquinones are widely distributed throughout the body, especially in organs and tissues with abundant blood flow [177]. Most anthraquinones are conjugated to glucuronides or sulfates, which are excreted through bile or urine.

To improve the bioavailability of quinones, nanotechnology has been widely used in recent years. By encapsulating quinones in nanoparticles, liposomes, or polymeric carriers, their solubility, absorption, and in vivo stability have been significantly improved. Emodin-loaded Poly-PEGMA-DMAEMA-MAM nanoparticles have shown efficacy in alleviating diabetic neuropathic pain by inhibiting purinergic P2X3 receptor expression, reducing TNF-α levels, and suppressing ERK1/2 activation in the dorsal root ganglia of type 2 diabetic rats [178]. Additionally, rhein-chitosan in situ hydrogel significantly accelerated wound healing in diabetic mice over a short period of two weeks [179]. This approach utilized the unique properties of hydrogels to provide a moist environment conducive to healing while delivering therapeutic agents effectively. In order to improve the in vivo utilization of CoQ10, novel CoQ10 formulations have been studied, such as CoQ10 nano-liposomes, lipid-CoQ10 conjugate nanodispersion (BPM31510), multicomposite CoQ10 terclatrate (Q-TER), CoQ10/b-cyclodextrin complexes, and nano-micellar CoQ10 formulations (Ubisol-Q10 and micellular formation with caspofungin), among others [25]. Formulations have been shown to have higher delivery efficiencies in vitro, and some of them have been shown to provide better CoQ10 bioavailability in vivo [180]. Intravenous administration of some formulations is particularly attractive for overcoming the poor and variable absorption of oral CoQ10 [181]. This method allowed for direct delivery into the bloodstream, bypassing gastrointestinal barriers and enhancing systemic availability. Controlled release systems have also been developed to optimize the pharmacokinetics and therapeutic efficacy of quinones. For instance, pH-sensitive polymeric nanoparticles have been engineered to release their quinone payloads specifically at the target site, enhancing local concentrations and minimizing systemic side effects [182]. Furthermore, stimuli-responsive delivery platforms, such as temperature or redox-sensitive carriers, enable the controlled release of quinones in response to specific physiological conditions, improving therapeutic precision [183]. In addition, traditional Chinese medicine (TCM) theory mentions that drug–drug interactions can enhance the absorption of quinones, such as that the components in wine-steamed rhubarb can accelerate the hydrolysis reaction through the metabolism of ethanol, which improves its bioavailability [184]. Combining quinones with bioavailability enhancers, such as bile salts, surfactants, or dietary lipids, has proven to be effective. Vitamin K1, for example, is better absorbed when ingested with vegetable oils rather than as part of raw vegetables [176].

In conclusion, although quinones show great promise in the treatment of metabolic diseases, especially diabetes mellitus, their low bioavailability remains an urgent challenge. In recent years, strategies such as nanotechnology, liposomes, self-emulsifying systems, chemical modifications, and eutectic technologies have made significant progress in enhancing the solubility, absorption, and in vivo stability of quinones. Future studies should continue to focus on the optimization of delivery systems and the assessment of long-term safety with a view to achieving a wider range of clinical applications for quinones.

## 8. Limitations and Prospects

Quinones, such as CoQ10, RC, VK1/2, and emodin, show potential health benefits, particularly in regulating blood sugar levels and managing diabetes. However, research on their mechanisms of action still faces a series of limitations and challenges. Quinones can regulate blood glucose balance by modulating mitochondrial function, inflammation, lipid profile, gastrointestinal absorption, etc., but the specific molecular mechanisms are still not fully understood. A systematic mechanistic framework has not yet been established, and there are still some unknown mechanisms that need to be discovered and validated. Multi-target mechanisms of action are a major feature of natural product interventions with active molecules, complicating the study of their precise mechanisms of action [185].

PQQ, as a potent antioxidant and mitochondrial function modulator, has demonstrated the ability to lower blood glucose levels and improve insulin sensitivity in animal experiments by enhancing mitochondrial function and inhibiting PTP1B enzyme activity. However, there are currently few clinical trials in humans, with research primarily focused on cognitive function and antioxidant effects, leaving limited direct clinical evidence for its use in diabetes treatment [186]. Meanwhile, CQ10 has shown significant anti-diabetic effects in clinical studies. Eight clinical trials have revealed that CQ10 could improve endothelial function, thereby reducing the occurrence of diabetic complications such as vascular lesions and nephropathy. Additionally, two double-blind placebo-controlled trials have, respectively, confirmed the significant improvement of diabetic neuropathy and retinopathy symptoms with CQ10 supplementation [187]. In studies on vitamin K, a 2 h oral glucose tolerance test demonstrated that vitamin K supplementation could effectively reduce blood glucose and insulin levels in prediabetic populations [188]. While these findings are promising, much of the current research on quinones’ glucose-lowering mechanisms is based on in vitro experiments and animal models, limiting their broader clinical applicability. Therefore, large-scale rigorously designed clinical trials are needed to verify its efficacy, long-term effects, and safety [189]. These clinical data will help to study individualized treatment regimens in different diabetes subtypes, age groups, genetic backgrounds, lifestyles, and diets, and to more effectively assess the possible toxic side effects of long-term use. In addition, the low bioavailability of quinones, which is determined by their chemical structure, limits their hypoglycemic effect. Therefore, future research may need to focus on developing effective drug delivery systems or on improving their biological properties.

The natural sources of quinones are diverse and include a wide range of vegetables, fruits, herbs, animal and marine products, and fermented products. The chemical structures are also highly diverse, and this diversity increases the complexity of quinones research and makes it difficult to develop uniform research standards. Quinones are usually found in low concentrations in the natural product, and there are many challenges in their extraction and standardization, and the extraction process needs to be further explored to be more efficient, economical, and environmentally friendly [190]. In addition, the stability problem of quinones is also a focus that should not be ignored, such as the photosensitivity problem of some quinones, such as RC and VK1 [50,191].

**Figure 6:**
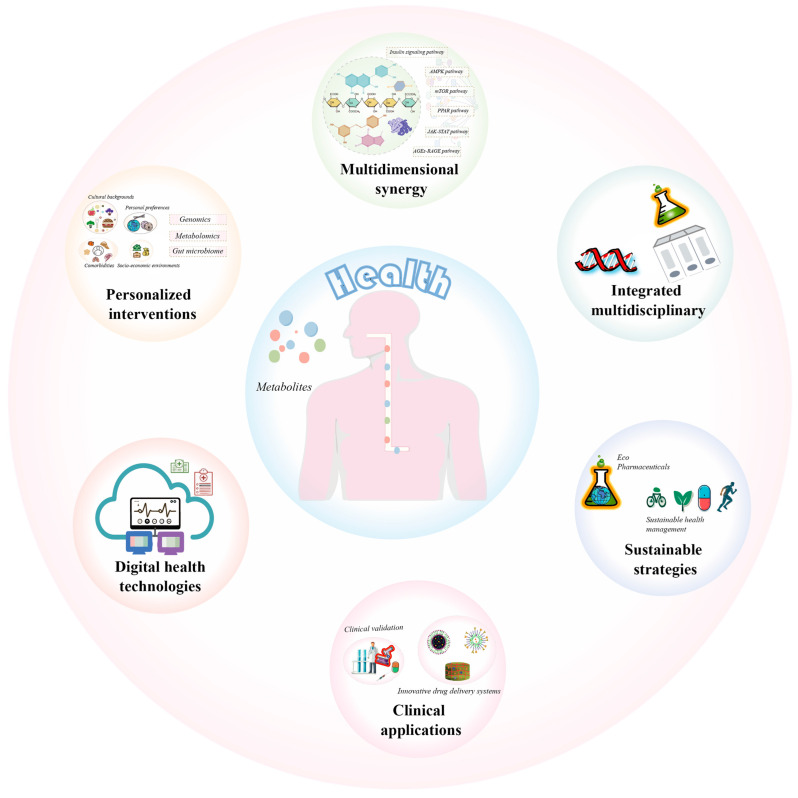
Multidimensional systematic research model for health control.

In diabetes prevention and treatment research, a multi-dimensional systematic model of health control can be proposed (Figure 6). Unlike traditional research that focuses on single components, this model highlights the multifaceted interactions of bioactive ingredients in natural products. By studying the synergistic effects of different natural product chemical compositions, revealing how they work together through multi-target mechanisms to influence physiological processes such as glucose metabolism, lipid metabolism, and inflammatory response, thereby improving the prevention and treatment of diabetes more comprehensively [192]. And one core of this research model lies in the realization of personalized health interventions. Due to the wide range of people with diabetes, their cultural backgrounds, personal preferences, comorbidities, and the socio-economic environments in which they live, a “one-size-fits-all” approach to prevention and treatment is impractical [193]. Moreover, considering the significant differences in the biological effects of natural products in different individuals, it is possible to tailor health interventions in combination with genomics, metabolomics, and gut microbiome technologies to achieve precision treatment [194]. This approach not only maximizes the therapeutic effect of natural products, but also improves the overall effect of the intervention by optimizing the combination and dosage of active ingredients to overcome fluctuations in effect due to individual differences. In addition, integrated interdisciplinary research and the application of digital health technologies are important directions to promote the development of this research model. The in-depth integration of food science, nutrition, pharmacy, metabolomics, gut microbiology, and information technology will provide more systematic and precise theoretical support for the application of natural products in diabetes nutrition prevention and treatment [195]. With the help of big data analysis and digital health platforms, the dynamic monitoring and real-time analysis of individual health data can be realized, thus continuously optimizing nutritional intervention strategies and ensuring more accurate and personalized treatment plans [196]. Ultimately, with the development of this model, the application of natural products in health intervention for diabetes and other chronic diseases will be more systematic and personalized. The model also advocates green and sustainable health intervention strategies, emphasizing the use of natural products to lower blood sugar through green pharmaceutical technology while promoting a healthy lifestyle, such as a balanced diet and moderate exercise, to support the long-term management and prevention of diabetes. At the same time, combining the bioactive mechanisms of natural products with modern drug development and innovative drug delivery systems (e.g., nano-delivery technology) will further enhance the effectiveness of natural products in clinical treatment and open up a broader prospect for diabetes prevention and treatment.

Expanding on this multi-dimensional approach, quinones hold significant promise beyond diabetes, with emerging applications in diverse health domains. Their inherent antioxidant, anti-inflammatory, and mitochondrial-modulating properties position them as versatile candidates for cancer therapy (e.g., tumor-targeted prodrugs and chemosensitization), neurodegenerative disease mitigation (via mitochondrial biogenesis and ROS scavenging), and cardiovascular health (reducing arterial calcification and lipid dysregulation) [5,197,198]. In antimicrobial resistance, quinones like juglone disrupt biofilms, while in autoimmune disorders, compounds such as emodin suppress NF-κB-driven inflammation [146,199]. Their role in anti-aging, particularly in combating sarcopenia and metabolic decline through mitochondrial support, further underscores their broad utility [31]. To optimize these applications, personalized strategies integrating genomics, metabolomics, and gut microbiome profiling could tailor quinone-based interventions to individual biological variability, enhancing efficacy and minimizing toxicity. Innovations like nano-delivery systems may overcome bioavailability challenges, while digital health platforms could enable the real-time monitoring of therapeutic responses. Interdisciplinary collaboration—spanning pharmacology, food science, and green chemistry—will drive sustainable production methods and synergistic formulations, aligning with the model’s emphasis on eco-friendly solutions. By embedding quinones within this systematic framework, future research can unlock their potential across oncology, neurology, and infectious diseases, advancing precision medicine and holistic health strategies.

## 9. Conclusions

Quinones in natural products have received much attention for their potential in hypoglycaemic activity. Despite their abundant sources and diverse classes, they still face major challenges in terms of bioavailability and toxicity. In recent years, the bioavailability and toxicity of these compounds have been significantly improved through nanotechnology, self-emulsifying systems, and chemical modifications, offering more possibilities for their applications. Future studies need to continue to explore the targeted delivery and multicomponent synergistic effects of quinones while devoting more attention to their toxicity and long-term safety assessment in order to promote their practical applications in diabetes treatment while promoting the formulation of dietary recommendations, the development of supplements or incorporation into drug therapies, or providing specific recommendations for future studies or clinical trials.

## Figures and Tables

**Table 1:** Anti-diabetic effects of quinones in vitro or in vivo.

Num.	Compound	Structure	Source	Research Model	Antidiabetic Effects	Reference	(Q/SQ^•−^)/mV
1	Coenzyme Q10	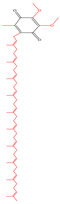	Corn, peanut, sardines, animal offal, and so on.	STZ-induced rat.	IGF-1 ↑, eNOS ↓, myocardial relaxation ↑, ROS ↓, mitochondrial function ↑.	[29]	−230
2	Thymoquinone	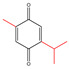	*Nigella sativa* L.	STZ-induced diabetes rats, diabetic mice during gestation and lactation.	Serum glucose ↓, serum insulin level ↑, body weight ↑; TAC ↑, MDA ↓, COX-2 ↓, SOD ↑, GST ↑, GPx ↑, CAT ↑, GSH ↑; IL-1β ↓, IL-6 ↓, TNF-α ↓.	[30,31]	−70
3	Embelin	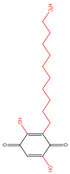	Fruit of *Embelia basal.*	α-glucosidase activity, high-fat diet (HFD) + streptozotocin (STZ) diabetic rats.	α-Glucosidase ↓; Body weight ↑, plasma glucose ↓, HbA1c ↓; IL 6↓, TNF α ↓; GSH ↑, SOD ↑, CAT ↑, MDA ↓; TC ↓, TG ↓, VLDL ↓, LDL ↓, HDL ↑.	[32]	-
4	Pyrroloquinoline quinone (PQQ)	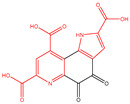	Fermented soy beans (natto), tea, green peppers, parsley, kiwi fruit, and human milk.	Diabetic KK-A(y) mice, diabetic UCD-T2DM rats, mouse C2C12 myotubes, HepG2 cells, brain of STZ-induced diabetic mice.	Regulate CREB-PGC-1α pathway; MDA ↓, GSH ↑, SOD ↑, CAT ↑, GPx ↑, LPO ↓, LOOH ↓; Regulate PTP1B/IRS-1/GLUT4 pathway; AGEs formation ↓, RAGE expression ↓; Regulate NF-κB/pyroptosis pathway, TNF-α ↓, IL-6 ↓, IL-1β ↓.	[4,33]	90
5	Rhinacanthin C	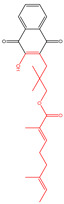	*Rhinacanthus nasuta* Kurz.	α-glucosidase inhibition, PTP1B inhibition, STZ-induced diabetic rats, 3T3 L-1 cells, STZ–nicotinamide induced male diabetic rats.	α-glucosidase ↓; PTP1B ↓; PPARγ ↑; FBG ↓, HbA1c ↓; LPO ↓, MDA ↓, SOD ↑, CAT ↑, GPx ↑; HDL ↑, TC↓, LDL ↓, VLDL ↓, HOMA-IR ↑; RAGE ↓, regulate NF-κB signaling pathway, TNFα ↓, Ikkβ ↓, caspase-3 ↓; GLUT4 translocation ↑, GLUT2 translocation ↑, glucose uptake ↑; AST ↓, ALT ↓, BUN ↓, creatinine ↓; G6PDH ↑, GDH ↑, SDH ↑; GK ↑, PFK ↑, PK ↑.	[34,35]	-
6	Plumbagin	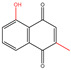	*Diospyros kaki* L., *Plumbago zeylanica* L.	STZ induced diabetic rats, high-glucose-induced HTR8/SVneo cell.	TGFβ1 via Nox4 pathway ↓; Glucose uptake ↑, GLUT4 translocation ↑; Regulate AKT/mTOR pathway; Bcl-2 ↑, Bax ↓, cleaved caspase-3 ↓, cleaved caspase-9 ↓.	[35,36,37]	−156
7	Shikonin	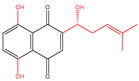	*Lithospermum erythrorhizon* SIEBOLD & ZUCC.	PTP1B inhibition, aldose reductase inhibition, L6 myotubes, 3T3-L1 cells.	PTP1B↓; Aldose reductase↓; FABP4↓, LPL↓, SREBP1C↓, PPARγ↓, C/EBPα↓; Glucose uptake↑, Akt↑, GLUT4 translocation↑.	[35,38]	-
8	Emodin	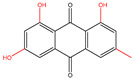	*Rheum palmatum*, *Polygonum cuspidatum*, *Polygonum multiflorum.*	PTP1B inhibition, aldose reductase inhibition, α-Glucosidase inhibition, DPP-4 inhibition, high-fat-diet-fed STZ- induced C57/BL6J diabetic mice, C2C12 myotubes, 3T3-L1 adipocytes, STZ- induced diabetic rats.	PTP1B↓; Aldose reductase↓; α-Glucosidase↓; DPP-4 ↓; PPARγ ↑; Regulate AKT/GSK-3β signaling pathway; Regulate the p38 MAPK pathway; FBG ↓, Glucose uptake ↑, translocation of GLUT2 and GLUT4 ↑; AGEs ↓, 11β-HSD1 ↓; Perilipin ↑.	[8,39,40]	−460
9	Aloe-emodin	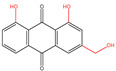	*Cassia occidentalis*, *Rheum palmatum* L., *Aloe vera*, *Polygonum multiflorum* Thunb.	PTP1B inhibition, α-Glucosidase inhibition, high glucose induced RIN-5F and L6 myotubes cells.	α-Glucosidase ↓; PTP1B ↓; Glucotoxicity ↓, ROS ↓; IL-1β ↓, IFN-γ ↓; Bax ↓, Fas ↓, Fadd ↓, caspase-3 ↓, Bcl-2 ↑, regulate NF-κB signaling pathway.	[8,39]	−405
10	Chrysophanol	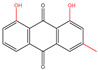	*Radix et Rhizoma Rhei*, *Cassia obtusifolia* L., *Polygonum multiflorum*, *Aloe vera*, *Senna septemtrionalis.*	PTP1B inhibition, α-Glucosidase inhibition, DPP-4 inhibition, STZ-induced diabetic mice, high-fat diet-induced obese mice.	α-Glucosidase ↓; PTP1B ↓; DPP-4 ↓; FBG ↓, activate SIRT6/AMPK signaling pathway.	[8,39]	−425

↑ represents an increase in expression level; ↓ represents a decrease in expression level; STZ, streptozotocin; HFD, high-fat diet; PTP1B, protein tyrosine phosphatase 1b; IGF-1, insulin-like growth factor 1; eNOS, endothelial nitric oxide synthase; ROS, reactive oxygen species; TAC, total antioxidant capacity; MDA, malondialdehyde; COX-2, cyclooxygenase-2; SOD, superoxide dismutase; GST, glutathione S-transferase; GPx, glutathione peroxidase; CAT, catalase; GSH, glutathione; IL-1β, interleukin 1beta; IL-6, interleukin-6; TNF-α, tumor necrosis factor alpha; HbA1c, hemoglobin A1c; TC, total cholesterol; TG, triglycerides; VLDL, very low-density lipoprotein; LDL, low-density lipoprotein; HDL, high-density lipoprotein; LPO, lipid peroxidation; LOOH, lipid hydroperoxides; IRS-1, insulin receptor substrate 1; GLUT4, glucose transporter 4; GLUT2, glucose transporter 2; PPARγ, peroxisome proliferator-activated receptor γ; HOMA-IR, homeostatic model assessment for insulin resistance; RAGE, receptor for advanced glycation end; AGEs, advanced glycation ends; NF-κB, nuclear factor kappa B; AST, aspartate aminotransferase; ALT, alanine aminotransferase; BUN, blood urea nitrogen; G6PDH, glucose-6-phosphate dehydrogenase; GDH, glutamate dehydrogenase; SDH, sorbitol dehydrogenase; GK, glucokinase; PFK, phosphofructokinase; PK, pyruvate kinase; Bcl-2, B-cell lymphoma-2; Bax, Bcl-2-associated X; AKT, protein kinase B; FABP4, fatty acid binding protein 4; LPL, lipoprotein lipase; SREBP1C, sterol regulatory element binding transcription factor 1; PPARγ, peroxisome proliferator-activated receptor gamma; C/EBPα, CCAAT/enhancer binding protein alpha; GSK-3β, glycogen synthase kinase 3 beta; 11β-HSD1, 11β-hydroxysteroid dehydrogenase type 1; IFN-γ, interferon-gamma; Fas, fatty acid synthase; Fadd, Fas associated via death domain; SIRT6, sirtuin 6; FBG, fasting blood glucose; AMPK, AMP-activated protein kinase.

**Table 2:** Dietary sources of quinones and their content.

Source Category	Specific Food/Organism	Quinone Type	Content (Examples)	Ref.
Plant Sources	Spinach, Kale, Broccoli, Lettuce, Cabbage, Vegetable oils, Green powdered tea	Vitamin K1 (VK1)	Spinach: 240–1220 µg/100 g Kale: 250–1139 µg/100 g Soybean oil: 180 μg/100 g Green powdered tea: 28.54–32.44 μg/g	[50,58]
	Parsley, Green Pepper, Spinach, Kiwi fruit, Papaya	PQQ	Parsley: 34.2 ± 11.6 ng/g Green pepper: 28.2 ± 13.7 ng/g Kiwi fruit: 27.4 ± 2.64 ng/g	[52]
	Black Cumin Seeds (Nigella sativa)	Thymoquinone	18–24% in seed oil	[57]
Animal and Marine	Heart, Liver, Shoulder, Sirloin, Thigh, Tenderloin, Fish	CoQ10	Beef heart: 113.3 mg/kg Pork heart: 118.1–282 mg/kg Chicken liver: 116.2–132.2 mg/kg Herring heart: 120.0–148.4 mg/kg	[51]
	Milk	PQQ	Breast milk: 140–180 ng/ml	[59]
	Butter	Vitamin K2 (VK2)	Butter: 15 µg/100 g	[5]
	Wakame, Laver	VK1	Wakame: 12,930 ng/g Laver: 4130 ng/g	[60]
Fermented Sources	Natto	PQQ, VK2	PQQ: 61.0 ± 31.3 ng/g VK2: 10 µg/g	[13,52]
	Parmesan cheese, Emmental cheese, Gouda cheese	VK2	Parmesan: 7.1–76.5 µg/100 g Emmental, 43.3 μg/100 g	[5]
	Pickles	VK2	Menaquinone-7: 5.55–14.48 µg/100 g Menaquinone-4: 1.00–4.63 µg/100 g	[61]

## Data Availability

Data sharing is not applicable.
